# Protective factors against suicide attempt in Iranian Kurdish women: a qualitative content analysis

**DOI:** 10.1186/s12888-023-04544-y

**Published:** 2023-01-21

**Authors:** Saeed Ariapooran, Mehdi Khezeli, Parisa Janjani, Hamid Jafaralilou, Sajad Narimani, Maryam Mazaheri, Mohsen Khezeli

**Affiliations:** 1 Department of Psychology, Malayer University, Malayer, Iran; 2 Social Development and Health Promotion Research Center , Health Institute Kermanshah University of Medical Sciences, Kermanshah, Iran; 3 Cardiovascular Research Center, Health Institute Kermanshah University of Medical Sciences, Kermanshah, Iran; 4 Department of Public Health, Khoy University of Medical Sciences, Khoy, Iran; 5 Social Determinants of Health Research Center, Ardabil University of Medical Sciences, Ardabil, Iran; 6 Department of Social Medicine and Family, School of Medicine, Dezful University of Medical Sciences, Dezful, Iran

**Keywords:** Protective factors, Qualitative study, Resilience, Social support, Suicide

## Abstract

**Background:**

A proper understanding of the protective factors against suicide attempt can provide the basis for planning preventive interventions. This study aimed to identify protective factors against suicide attempt among women.

**Methods:**

This qualitative study was conducted in Kermanshah, Iran between January and May 2021. Participants were 20 Kurdish women, survivors of suicide attempt, selected by purposive sampling method. The data collection method was face-to face and audio-recorded semi-structured interview. Qualitative data analysis was done according to Diekelmann 7-step approach.

**Findings:**

According to the results, the main topic was protective factors against suicide attempt in women, with two categories; “Individual factors” and “Social factors”. “Individual factors” had five subcategories including coping strategies, reasons for living, resilience, religious beliefs, and fear of death, and “social factors” had two subcategories including social support and effective communication.

**Conclusion:**

This study showed that women who intend to commit suicide may encounter some individual and social factors that play a protective role against suicide. It is recommended to identify and strengthen these protective factors for the effectiveness of suicide prevention interventions.

## Background

Suicide is a major global health problem, however, low- and middle-income countries account for a greater proportion of cases [1]. Attempted suicide is defined as a self-initiated sequence of behaviors by an individual who, at the time of initiation, expected that the set of actions would lead to his or her own death [2]. According to the report of the World Health Organization (WHO), the worldwide number of suicide cases in 2019 was 703,000, representing 9 deaths per 100,000 people [3]. Also, the age-standardized suicide rate for all ages (per 100,000) in Iran has been reported as 5.5 [3]. The results of a study showed that the average annual mortality rate in Kermanshah province during an 11 years period was 15.77 per 100,000, and hanging (48.1%) and self-immolation (47.8%) were common methods of suicide in men and women, respectively [4]. Another study showed that Kermanshah province has the second highest suicide rate in Iran after its neighboring province of Ilam [5]. We conducted this study on women because studies have shown that suicide attempt in Kermanshah province is significantly higher in women than in men [6, 7].

To investigate suicide attempt, special attention should be paid to the two terms of risk factors and protective factors [1]. Risk factors are those that show whether a person, a community or a population is particularly vulnerable to suicide [8]. There is a consensus that suicidal behavior is not a single-caused behavior, and there is agreement that this behavior clearly results from a complex interaction of many different factors [9]. Accordingly, many theories and models have been presented to explain suicidal behaviors. For example, interpersonal theory of suicide posits that mental pain and communication difficulties (marked by thwarted belongingness and perceived burdensomeness) are factors that work together to motivate a person to engage in suicidal behavior [10]. A broad wider model; the integrated motivational– volitional (IMV) model of suicidal behavior postulates that suicide attempt is the result of the interaction and effects of multiple risk factors at three levels: pre motivational phase includes diathesis, environment, and life event, motivational phase includes psychosocial factors that cause suicidal thoughts and intent, and volitional phase contains risk factors that transit suicidal ideation/intent to action [11]. The cultural theory and model of suicide also believes that many cultural factors are involved as risk factors in suicidal behaviors [12]. In the case of Iran, factors such as marital discord, love and strong emotional tendencies, educational failures, traditional Iranian patriarchal culture, economic constraints, and work problems are introduced as predisposing factors of suicidal behaviors [13–15].

As the subject of our study, there are protective factors that protect people from the risk of suicide. While many interventions focus on reducing suicidal risk factors, it is equally important to strengthen factors that increase resilience and connectedness and protect individuals against suicidal behaviors [1]. Rodgers (2011), identifies four main factors protecting against suicide attempts: mental health care, connectedness, problem-solving skills, and contact with health care providers [16]. Meaning in life is introduced a protective factor against suicidal tendencies through the creation of protective responses such as optimism, planning for the future, life satisfaction, and assertiveness in dealing with problematic situations [17]. The results of a cross-national study confirmed that social support is a significant protective factor against suicide in men and women [18]. Especially, women who receive a high level of social support from the family are less likely to attempt suicide than those with low social support [19]. The results of a comparative study in Iran showed that intrinsic religiousness, support from friends, and problem-focused coping strategies were significant protective factors against suicide attempts [20]. Feeling hopeful, feeling happy, and ringing for a general chat were also significant protective factors against suicidal behaviors in help-seekers [21].

Some documents have suggested that in preventive interventions to reduce suicidal behaviors, it is better to emphasize strategies that promote protective factors against suicide in people [8, 22, 23]. Given that suicide attempt is a global public health problem that is highly dependent on cultural, social, and psychological contexts in different parts of the world, achieving a correct understanding of factors related to suicide attempt, especially protective factors, requires qualitative studies [24]. Therefore, the present study was conducted to identify protective factors against suicide attempt in women of Kermanshah, a region in western Iran.

## Methods

The present study was a qualitative study with content analysis method that was conducted to investigate the protective factors of women against suicide attempt.

### Participants and sampling

This qualitative study was conducted in the city of Kermanshah in western Iran between January and May 2021. In this study, a purposeful sampling strategy was used to select participants. Inclusion criteria were: female gender, age between 18 and 59 years, having at least once a history of suicidal attempt during the year before the study, and expressing informed consent to participate in the study. The exclusion criterion was withdrawal from the study at any time of the study. In qualitative research, the sample size depends on data saturation that is related to the extent to which the new data repeats what was stated in the previous data [25]. Accordingly, in the present study, data saturation was obtained by interviewing 20 women who had attempted suicide at least once during the year before the study. To select the samples, first a list of women who had attempted suicide was prepared. Then women were called and after presenting the objectives of the study, they invited to participate in the study. In case of satisfaction, the data collection using interview was carried out. The setting of the study and the place of data collection was the counseling room of the mental health unit of Kermanshah health centers.

### Data collection

Individual interviews were conducted based on an interview guide that had open-ended questions about the various dimensions of the topic, i.e., suicidal behaviors and protective factors. In the interview guideline, questions were asked about the history of suicidal thoughts, intention and attempt, and the factors preventing the suicide attempt in case of the intention to attempt. Since the interview questions were open-ended, according to the answers of the interviewees, follow-up questions were asked for further explanation and clarification of the protective factors according to each case. Demographic questions were raised at the beginning of the interview guide. This interview guide was compiled using a text review by the research team focusing on key points. The guideline questions were based on the general goal of determining what factors often prevented the interviewees from translating their thoughts or intentions into suicide. The data collection method was face to face semi-structured interview.

### Procedures

The interviews were conducted after explaining the purpose of the study and obtaining informed consent from the participants. All interviews were conducted by a female researcher and audio recorded. All interviews were conducted in Kurdish language to gain a mutual understanding of the parties and to gain a deeper understanding of the phenomenon under study.

### Analysis

We used the method of Diekelmann et al., in analyzing the text of narratives [26]. Accordingly, in the first stage, the content of the interviews transcribed verbatim in Persian immediately after each interview. Notation that the interviewer created during the interview about the participants' body movements and postures were also used in data analysis at this stage. It is necessary to state that the analysis process was carried out separately by three researchers, including first author, a psychologist, second author, a health promotion specialist, and last author, a sociologist. In step 2, each individual text was summarized in explanatory sections. At this stage, meaningful statements or excerpts from texts that were interpreted as semantic units, such as repeated words, thoughts and beliefs, or actions, were identified. Then these semantic units were labeled in the form of different codes. In the third step, each analyst made a re-evaluation of the extracted findings. For this purpose, the researchers referring the main text and conversations of the interviewees reached an individual conclusion regarding semantic units and codes. In the fourth stage, the texts produced in the previous stages of analysis were aggregated. The purpose of this step was to identify the common findings of the three analysts. In this way, the three researchers shared the extracted semantic codes to identify the similarities or contrasts of meaning in the participants' conversations. In the fifth stage, with the participation of three researchers, subcategories including coping strategies, reasons for living, resilience, religious beliefs, and fear of death, social support, and effective communication were defined and organized in two categories of individual protective factors and social protective factors. Also, protective factors against suicide attempt, which surrounded the above two categories was also defined as the main topic. In the next step any disagreement between the three researchers was resolved by the third author of the article. In the last step, after validating the data, which is described in the next paragraph, the results were summarized and confirmed among the research team. Excerpts from the participants' conversation were also included in the final written report.

### Trustworthiness

In this study, the four criteria of Lincoln and Guba including credibility, transferability, confirmability, and dependability were used to check the validity of the data [27]. To ensure the credibility of the findings, we used debriefing by peer and member check methods. In the case of peer debriefing, we had a request to peers who were not researchers on the study but had critique expertise in the field of suicide including specialists in sociology and suicidality. The research team committed themselves to adhere to the topics of the peers and take their opinions into account in the final conclusions. In order to member check, a summary of the interview was returned to the participants to confirm or refute the accuracy of the researcher’s perception. In cases of disagreement between the perception of researchers and the opinion of the participant, we tried to reach a common understanding about the subject through interaction with participants. To improve the transferability of the findings, purposeful and heterogeneous sampling was used in terms of education, type of suicide attempt, age, and marital status. Audit trial and peer review were used to control the dependability of data. Our research team with different expertise reviewed, revised, and confirmed all encoded data and determined subcategories and categories to confirm the confirmability of the data.

### Ethical aspect of the study

Confidentiality of information was guaranteed to all participants, and written informed consent form was obtained from all of them. This study received ethics approval from the Research Ethics Committee Kermanshah University of Medical Sciences (No. IR.KUMS.REC.1399.602).

## Findings

Twenty women with a history of suicide attempt participated in this study. The mean and standard deviation of the participants' age was 33 ± 6.54 years. Most of the participants were housewives and literate.

## Concepts identified in interviews

In this study, the main topic was protective factors against suicide attempt in women, which included two categories of "individual protective factors" and "social protective factors", along with seven subcategories (Table 1).

**Table 1:** Main topic, categories, and subcategories extracted from the interviews

Main topic	Categories	Subcategories
**Protective factors against suicide attempt**	***Individual protective factors***	*Coping strategies*
		*Reasons for living*
		*Resilience*
		*Religious beliefs*
		*Fear of death*
	***Social protective factors***	*Social support*
		*Effective communication*

- **1. Individual protective factors: **This category consists of five subcategories, including: "coping strategies", "reasons for living", "resilience", "religious beliefs", and "fear of death".
- ***1.1*** **Coping strategies:** Participants mentioned some activities that could have stopped the thought of suicide including going to relatives’ house or market, praying, drinking tea, doing housework, calling their favorite people, watching TV and reading book.

Participant # 14 "*When my husband was arguing with me and I was thinking about suicide, I tried to control my stress and anxiety. Sometimes I would call my sister and talk about my problems".*

- ***1.2*** **Reasons for living:** One of the protective factors in this study was remembering the reasons for living when suicidal thoughts occur. Respondents stated that when they had doubts about suicide and survival, they tried to prevent attempting suicide by remembering the reasons for living. Some of these reasons were hope for the future and problem solving, raising and nurturing children, and focus on the positive aspects of life.

Participant # 11 "*I thought about* suicide *several times, but I only tried to attempt suicide once and in other cases, I gave up trying to attempt suicide, because in most of the time, I thought that life has many aspects to enjoy and continue such as my children and my family*".

In the case of raising and nurturing children, the presence of young children in the family was introduced as a reason for survival and preventing suicide by two women.

Participant # 4: "*I stopped trying to attempt suicide several times because of my little girl. Every time I thought about what would happen to this child after my death, I preferred to live."*

- ***1.3*** **Resilience:** Content analysis of the interviews showed that there were cases where women mentioned a personal characteristic called resistance to the temptation to attempted suicide, which we labeled it as resilience. Women stated that they had strong and justified reasons for attempting suicide, but by enduring problems and increasing the ability to deal with suicidal thoughts or intent, they were able to prevent suicide attempt in most cases.

Participant # 19 "*Before I attempted suicide, I tried to kill myself several times. In many cases, I told myself that I could go on with my life and not kill myself, and I told myself that I was strong and I can get over these problems*."

Participant # 14 *"I often believed in my own ability to deal with suicidal thoughts and endured difficulties."*

- ***1.4*** **Religious beliefs:** Some participants mentioned the protective role of religious beliefs. They cited religious beliefs such as the guilt of suicide in the Qur'an, attending religious meetings to strengthen faith, praying in times of trouble and thinking about suicide as factors in protecting against suicide attempts.

Participant # 5 "*I have refused to attempt suicide several times because I am a religious person. I know that in Islam, suicide is an unforgivable sin and a forbidden act. Although I attempted suicide last year, I still regret and I hope God forgives me*".

- ***1.5*** **Fear of death:** In this study, five participants identified the fear of death as one of the important factors in refusing from suicide. They believed that when they thought about the possible consequences of suicide, such as pain, suffering, and eventually death, they stopped attempted suicide.

Participant # 2 "" *I always had arguments with my husband. I wanted to burn myself to make my husband notorious. But I was afraid of dying. I was more afraid of dying by burning myself than anything else*".

- **2. Social protective factors:** In this study, the interviewees mentioned some protective factors that were related to others and in the social sphere. The women referred to some support from their husbands, children, other family members, friends and social contacts, which we categorized into two groups: "social support" and "effective communication".
- ***2.1*** **Social support:** Support from spouses, family members, relatives and friends were protective factors that protected women from attempting suicide. Some women emphasized that the support of their husbands, in times of crisis, was very promising and helpful. They stated that their spouses played an important role in preventing them from attempting suicide. A number of other interviewees also stated that the support of family members and friends prevented them from attempting suicide in times of hardship and in situations where they had suicidal thoughts.

Participant # 18 "" *When I was very depressed, I did not like life. My husband found that sometimes I think about suicide. He takes me on a trip, buys me gift, and comes home sooner. Every time I thought about suicide, his supports made me stop attempting suicide*."

- ***2.2*** **Effective communication:** In this study one of the effective social factors in protecting women with suicidal ideation against suicide attempt was effective communication. According to interviews, if women have larger and wider social networks, they are less likely to attempt suicide. The communication in the present study was mostly related to the person herself, family, relatives and friends. In two cases, however, they referred to broader social networks, such as contacting a counselor or attending a sports club.

Participant # 3: "*Whenever I had the thought of suicide, I would quickly go to the phone and call my sister or mother. I tried to talk about everything until time passed. I think being in contact with family can prevent the thoughts of suicide*".

## Discussion

In this study, the results showed that both individual and social factors have a protective role against suicide attempt. Protective factors are one of the most important factors both in preventing the formation of suicidal ideation and in people who have suicidal ideation or plan to attempt suicide. While many interventions are performed to reduce risk factors for suicide prevention, it is equally important to consider and reinforce the factors that protect individuals from suicidal behaviors [16]. In the present study, based on interviews and participants' opinions, we extracted five individual protective factors against suicide attempt, including problem-solving skills or coping strategies, reasons for living, resilience, religious beliefs, and fear of death. We also identified two social protective factors against suicide, including social support and effective communication.

There are limited studies that have comprehensively examined protective factors against suicide. Most of the studies have investigated some protective factors in different populations in a single or multi-subject manner, and less have focused on the classification of individual and social protective factors. Also, the conceptualization of protective factors through theories and models has rarely happened. In one of these works, IMV model has introduced individual and social protective factors including reasons for living, attainable positive future thinking, adaptive goal pursuit, belongingness and connectedness as motivational moderators [11]. A theory driven network analysis also concluded that optimism, resilience and goal re-engagement were protective factors against suicide ideation [28]. In this study, we tried to present a clearer picture of these issues by categorizing protective factors in the form of individual factors and social factors, which are discussed below.

Positive coping strategies protect people against suicide. Emotional stability, optimism about the future, and developed self-identity help a person cope with life's problems. Good self-esteem, self-efficacy, problem-solving skills, and help seeking skill in critical situations can mitigate the impact of stressors on suicidal behaviors [1]. In the strain theory of suicide, a defect in positive coping skills is introduced as a source of strain, leading translate the suicidal ideation to suicide attempt [29]. Zhang and Li (2013), found that women who attempted suicide had lower scores of coping and problem-solving skills [30]. Jobes et al. (2015), believe that by using the right problem-solving skills and problem-oriented coping strategies, individuals can moderate the trigger role of stressors and prevent impulsive suicidal behaviors [31]. According to the three-step theory of suicide and suicide strain theory, hopelessness is one of the predisposing factors for suicidal ideation [29, 32]. On the other hand, some type of coping strategies are tools that protect people against despair and suicidal behaviors [29]. It should be mentioned that not all coping strategies necessarily protect people against suicide. For example, Liang et al. showed that among coping strategies, self-distraction, substance abuse, behavioral disengagement, venting, and self-blame had a positive relationship with suicidality [33]. Another study has also showed that an excessive avoidable coping is likely to lead to suicidal behaviors [34].

Reasons for living such as hope for the future, having and nurturing children, and enjoying the positive aspects of life were individual protective factors against suicide attempt in this study. Prospective studies have shown that people who have fewer reasons to live are at greater risk for suicidal behaviors [30, 35]. As suggested by the IMV model, the reasons for living and wish to live in the motivational phase of the model can weaken the relationship between defeat and entrapment with suicidal ideation and intent [11]. Another study suggested that having reasons to live can protect people from suicidal behavior by weakening the relationship between depression and suicidal ideation [36]). Consistent with some studies [37, 38], we found that having a young child at home as a reason for living can be protective against suicidal behaviors. It seems that despite the inequalities and discrimination against women in the traditional society of Iran, some reasons for continuing life such as having a small child, keeping the family, and hoping for a better future temporarily protect women from suicide attempts in the present study.

Resilience was reported as an important personal protective factor against suicide attempt by women in this study. Resilience is a dynamic process that allows an individual to adapt to and overcome stress [39]. According to Johnson et al. (2010), resilience can weaken the process of suicidal ideation during the stressful life events [40]. Similar to the results of our study, a study in Iranian Kurdish women showed that resilience is one of the protective factors against suicidal ideation [41]. The buffer hypothesis suggests that for people who are at risk of suicidal behavior, resilience acts as a buffer and weakens or eliminates suicidal behavior [42]. Review studies have shown that improving resilience is one of the most key interventions to prevent or reduce suicidal behaviors [39, 43]. One of the ways of impacting resilience on reducing suicidal behaviors can be through reducing impulsivity [44]. In support of this opinion, Ram et al. (2019) found that in attempted suicide, cognitive flexibility and resilience are interrelated positively and inversely associated with impulsivity [45].

In the present study, several participants cited the role of religion as a protective factor against suicide attempts. Religion is likely to have a protective effect in a variety of ways such as valuing the right to life, and promoting social bonds, which may reduce the risk of mental disorders such as depression and antisocial behaviors [46]. In the Holy Qur'an, in Surah 4, verses 29 and 30, suicide is explicitly forbidden and the eternal punishment for suicide is burning in hell. Probably one of the reasons for the lower suicide rate in Muslims is that Islam is stricter about the sin of suicide than other religions [47]. In addition, due to the fact that suicide is forbidden in Islam, it is still considered a crime in many Islamic countries [48]. However, the social stigma of suicide in Muslim countries and the possibility of underreporting suicide rates should not be ignored [49]. Taking all considerations into account, this topic can be used in suicide prevention interventions in Islamic countries.

In the present study, some women mentioned the fear of death as a protective factor against attempting suicide. Despite the importance of the topic, not many studies have been conducted to examine the relationship between fear of death and suicide attempts, but the few available evidences have not reported contradictions about this relationship. The simultaneous presence of suicidal ideation and reduced fear of death creates a situation in which the suicidal tendency becomes active and turns into suicide attempt [10]. Britton and colleagues (2008), showed that patients who reported higher levels of fear of dying by suicide were less likely to experience suicidal thoughts [50]. A study has shown that the fear of death acts as a protective shield against suicidal behaviors [51]. The interpersonal theory of suicide suggests that the ability to overcome the fear of death and suicidal desire are prerequisites for attempting suicide [10].

In the present study, based on the content analysis, we recognized two social protective factors against suicide attempts, including social support and effective communication. Studies have shown that social support has an effective role in controlling or reducing suicidality [52]. Khezeli et al. (2019) showed that peer and family support reduces suicidal ideation, while family conflicts increase suicidal ideation and attempt in women [53]. Meadows et al. similarly showed that women who receive more family support are less likely to attempt suicide [19]. A study by Ariapooran and Khezeli in Kermanshah (2018), also showed that perceived social support in women was inversely related to suicidal ideation [41]. Some models and theories of suicide have provided justifications regarding the role of social support in modulating suicidal behaviors. In the Cry of pain model (CoP), "no rescue" as the third component of the model directly refers to the level of social support (or loneliness) and is an important factor in explaining suicidal and self-harming behaviors [54, 55]. The Schematic Appraisals Model of Suicide (SAMS) also suggests that perceived social support influences suicidal ideation through appraisals of defeat and entrapment [56]).

There is evidence that the intrapersonal and interpersonal quality communication can protect people from suicidal behaviors [57]. According to WHO in 2014, the risk of suicidal behaviors increases when people have severe communication conflict or disagreement. On the contrary, the continuation of intimate communication can increase people's self-esteem and act as a protective factor against suicide [1]. Positive and effective communication helps to deal with stress, reduce hopelessness and depression in the face of painful life events and increase people's resilience by increasing intimate relationships [58]. One type of effective communication is suicide communication, in which people share their suicidal thoughts or intentions with others. The results of a study have shown that communication with suicide is associated with a lower chance of suicide attempt in people [59]. Khezeli et al. (2019), in Iranian Kurdish women showed that the score of suicidal ideation was higher in those who had decreased effective communication [53]. Regarding the classification of social protective factors, it may seem that these two subcategories overlap. In this study and by analyzing the data, we found out that people viewed the tangible or perceived support they received from others as one of the protective factors against suicide attempts. However, there were also cases where support from others was not provided at first, but people actively sought a verbal or face-to-face communication. This may overlap slightly with social support, and this subcategory can also be defined as help-seeking behaviors. However, the research team concluded that effective self-initiated communication in this study can be classified separately from social support.

This was a qualitative study with a content analysis approach that was conducted on a sample of Kurdish women in Kermanshah, western Iran. The limitation of this study due to the nature of qualitative studies is that the generalization of the results cannot be considered with certainty. However, this study provided a good insight into the protective factors against suicide in a sample of Iranian Kurdish women. The strength of this study was the conduct of interviews in Kurdish language by the female interviewer to gain a deeper understanding of the subject and to communicate appropriately with the interviewees. Another strength of this study is conducting in-depth interviews and immersing the researcher in the data by reviewing them several times, which led to extracting, reporting and classifying the concepts hidden in women's experiences.

## Conclusions

The present qualitative study, showed that some individual factors such as coping strategies, reasons for living, resilience, religious beliefs, and fear of death, and two social factors including social support and effective communication have played a protective role for women against suicide attempt. Based on the results of this study, interventions are suggested to increase women's resilience and make them stronger psychologically, which include teaching coping strategies, strengthen religious beliefs, looking for the philosophy of existence and life, extending intra and interpersonal communications, and especially the promotion of social support, which does not require special planning and costs, and its importance should only be explained and highlighted for families.

## Data Availability

The datasets used and/or analyzed during the current study available from the corresponding author on reasonable request.

## Abbreviations

CoP: Cry of Pain
IMV: Integrated Motivational– Volitional
SAMS: Schematic Appraisals Model of Suicide
WHO: World Health Organization
