# Correction to: Protective factors against suicide attempt in Iranian Kurdish women: a qualitative content analysis

**DOI:** 10.1186/s12888-023-04710-2

**Published:** 2023-04-18

**Authors:** Saeed Ariapooran, Mehdi Khezeli, Parisa Janjani, Hamid Jafaralilou, Sajad Narimani, Maryam Mazaheri, Mohsen Khezeli

**Affiliations:** 1 Department of Psychology, Malayer University, Malayer, Iran; 2 Social Development and Health Promotion Research Center, Health Institute Kermanshah University of Medical Sciences, Kermanshah, Iran; 3 Cardiovascular Research Center, Health Institute Kermanshah University of Medical Sciences, Kermanshah, Iran; 4 Department of Public Health, Khoy University of Medical Sciences, Khoy, Iran; 5 Department of Nursing and midwifery, School of Nursing, Social Determinants of Health Research Center, Ardabil University of Medical Sciences, Ardabil, Iran; 6 Department of Health in Disasters and Emergencies, School of Health Management and Information Sciences, Iran University of Medical Sciences, Tehran, Iran; 7 Department of Social Medicine and Family, School of Medicine, Dezful University of Medical Sciences, Dezful, Iran

Following the publication of the original article [1], the authors would like to add an affiliation to Sajad Narimani. The added affiliation is given below.

Department of Health in Disasters and Emergencies, School of Health Management and Information Sciences, Iran University of Medical Sciences, Tehran, Iran.

The original article [1] has been corrected.
